# A longitudinal study of fecal calprotectin and the development of inflammatory bowel disease in ankylosing spondylitis

**DOI:** 10.1186/s13075-017-1223-2

**Published:** 2017-02-02

**Authors:** Eva Klingberg, Hans Strid, Arne Ståhl, Anna Deminger, Hans Carlsten, Lena Öhman, Helena Forsblad-d’Elia

**Affiliations:** 1 Department of Rheumatology and Inflammation Research, Sahlgrenska Academy at the University of Gothenburg, Guldhedsgatan 10A, S-413 46 Gothenburg, Sweden; 2 Department of Internal Medicine, Södra Älvsborgs Sjukhus, Borås, Sweden; 3 Department of Internal Medicine and Clinical Nutrition, Sahlgrenska Academy at the University of Gothenburg, Gothenburg, Sweden; 4 Department of Microbiology and Immunology, Sahlgrenska Academy at the University of Gothenburg, Gothenburg, Sweden; 5 Sweden School of Health and Education, University of Skövde, Skövde, Sweden; 6 Rheumatology Unit, Department of Public Health and Clinical Medicine, Umeå University, Umeå, Sweden

**Keywords:** Ankylosing spondylitis, Spondylarthritis, Inflammatory bowel disease, Fecal calprotectin, Crohn’s disease, Ulcerative colitis, Intestinal inflammation

## Abstract

**Background:**

Patients with ankylosing spondylitis (AS) are at increased risk of developing inflammatory bowel disease (IBD). We aimed to determine the variation in fecal calprotectin in AS over 5 years in relation to disease activity and medication and also to study the incidence of and predictors for development of IBD.

**Methods:**

Fecal calprotectin was assessed at baseline (*n* = 204) and at 5-year follow-up (*n* = 164). The patients answered questionnaires and underwent clinical evaluations. At baseline and at 5-year follow-up, ileocolonoscopy was performed in patients with fecal calprotectin ≥500 mg/kg and ≥200 mg/kg, respectively. The medical records were checked for diagnoses of IBD during the follow-up period.

**Results:**

Fecal calprotectin >50 mg/kg was found in two-thirds of the patients at both study visits. In 80% of the patients, fecal calprotectin changed by <200 mg/kg between the two measuring points. Baseline fecal calprotectin was positively correlated with Ankylosing Spondylitis Disease Activity Score based on C-reactive protein, Bath Ankylosing Spondylitis Disease Activity Index, Bath Ankylosing Spondylitis Functional Index, C-reactive protein, erythrocyte sedimentation rate, and fecal calprotectin at 5-year follow-up. The use of nonsteroidal anti-inflammatory drugs (NSAIDs) was associated with higher fecal calprotectin, and 3-week cessation of NSAIDs resulted in a drop of a median 116 mg/kg in fecal calprotectin. The use of tumor necrosis factor (TNF) blockers was associated with lower fecal calprotectin at both visits, but the users of TNF receptor fusion proteins had significantly higher fecal calprotectin than users of anti-TNF antibodies at 5-year follow-up. The 5-year incidence of Crohn’s disease (CD) was 1.5% and was predicted by high fecal calprotectin.

**Conclusions:**

Fecal calprotectin was elevated in a majority of the patients and was associated with disease activity and medication at both visits. CD developed in 1.5% of the patients with AS, and a high fecal calprotectin was the main predictor thereof. The results support a link between inflammation in the gut and the musculoskeletal system in AS. We propose that fecal calprotectin may be a potential biomarker to identify patients with AS at risk of developing IBD.

**Trial registration:**

ClinicalTrials.gov identifier: NCT00858819. Registered 9 March 2009. Last updated 28 May 2015.

## Background

Ankylosing spondylitis (AS), Crohn’s disease (CD), and ulcerative colitis (UC) are inflammatory diseases that have many common features. Several risk genes are shared between the spondylarthritides and inflammatory bowel disease (IBD), and the diseases can show coinheritance [1–4]. Patients with IBD can develop peripheral arthritis (10–20%), sacroiliitis (10–20%), and anterior uveitis (0.5–3%) [5–9]. Studies have revealed the presence of endoscopic and histologic gut inflammation in 40–60% of patients with AS and in 46% of patients with early spondylarthritis (SpA) [10–13]. The subclinical gut inflammation in AS is often localized to the colon or the distal ileum. Histologically, the inflammation has been divided into an acute form and a chronic form, the latter resembling CD and conferring an increased risk for subsequent development of IBD [14–16]. Approximately 5–10% of patients with SpA eventually develop IBD, with CD being more common than UC [17, 18]. There is a lack of knowledge about what predicts the development of IBD in AS, however, and clinical studies on the subject are rare [19].

Fecal calprotectin is an unspecific marker for gut inflammation. Calprotectin belongs to the family of calcium-binding calgranulins (or S100 proteins) and consists of heterodimers of the two proteins S100A8 and S100A9. Calprotectin is an abundant protein in the neutrophils, constituting up to 40–60% of the cytosolic protein content, and it is also found in gut epithelial cells, monocytes, and macrophages. The level of calprotectin in feces is proportional to the level of neutrophil inflammation in the gut [20]. Fecal calprotectin is clinically used to discriminate IBD from irritable bowel syndrome and correlates well with clinical, endoscopic, and histologic measures of disease activity in IBD [21, 22]. Fecal calprotectin is also increased in enteropathy caused by nonsteroidal anti-inflammatory drugs (NSAIDs) [23].

We have previously investigated the levels of fecal calprotectin in a cohort of 204 patients with AS. We found that 68% of the patients had elevated levels (>50 mg/kg) and that fecal calprotectin was associated with higher disease activity but not with gastrointestinal symptoms [24]. The same patients were then invited to be reexamined at a 5-year follow-up examination.

The aims of this 5-year prospective study on AS were (1) to investigate the intraindividual variations in fecal calprotectin over time in relation to disease activity, disease manifestations, and medication; (2) to study the 5-year incidence of IBD; and (3) to identify predictors for the development of IBD.

## Methods

### Patients

**Fig. 1:**
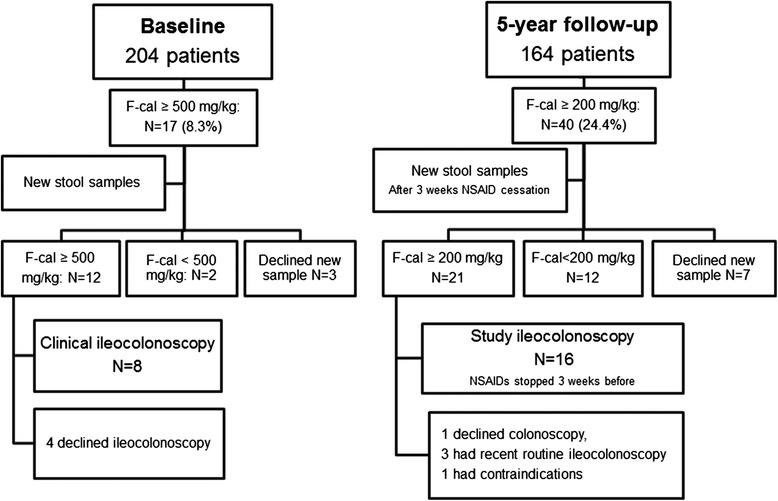
Flowchart of the study procedures. Ileocolonoscopies were performed at different thresholds of fecal calprotectin at baseline and 5-year follow-up. *F-cal* Fecal calprotectin, *NSAID* Nonsteroidal anti-inflammatory drug

All patients fulfilling the modified New York criteria for AS who were registered at the rheumatology clinics at Sahlgrenska University Hospital and the hospitals of Borås and Alingsås were invited to participate in the study in 2009, hereafter called *baseline* [24, 25]. Exclusion criteria were diagnosed IBD, psoriasis, dementia, pregnancy, and difficulties in understanding the Swedish language. Altogether, 204 patients were included at baseline and 164 (80%) patients were reexamined at the 5-year follow-up in 2014. All patients gave their written informed consent. The study was approved by the regional ethics committee in Gothenburg and was carried out in accordance with the Helsinki declaration. A flowchart of the enrollment of the patients and the study procedures is shown in Fig. 1. The patients underwent the same assessments at both visits, including physical examinations and responding to questionnaires. Blood and stool samples were collected. The medical records of all the included patients at baseline were checked for ileocolonoscopies, episodes of gut inflammation, or diagnoses of IBD.

### Questionnaires

The Ankylosing Spondylitis Disease Activity Score based on C-reactive protein (ASDAS_CRP_), Bath Ankylosing Spondylitis Disease Activity Index (BASDAI), Bath Ankylosing Spondylitis Patient Global Score, and Bath Ankylosing Spondylitis Functional Index (BASFI) were used to assess disease activity and physical function. Back mobility was measured for the calculation of Bath Ankylosing Spondylitis Metrology Index (BASMI) [26].

### Stool samples

Stool samples were collected by the patients and sent to the laboratory, where the samples were immediately frozen. After thawing of the samples, fecal calprotectin was analyzed using an enzyme-linked immunosorbent assay (ELISA) kit (Bühlmann Laboratories AG, Schönenbuch, Switzerland). The same ELISA kit and procedures were used at baseline and at 5-year follow-up. In the general population, fecal calprotectin is <50 mg/kg, according to the manufacturer of the kit.

### Blood samples

Blood samples were analyzed for hemoglobin, erythrocyte sedimentation rate (ESR), and C-reactive protein (CRP) using standard laboratory techniques. Serum calprotectin was analyzed at baseline by ELISA (PhiCal; Immundiagnostik AG, Bensheim, Germany) following the manufacturer’s instructions. According to the manufacturer, the normal range for serum calprotectin is 500–3000 ng/ml.

### Ileocolonoscopy

At baseline, all patients with fecal calprotectin ≥500 mg/kg were asked to send in a new stool sample. NSAID use was not stopped. If their fecal calprotectin remained ≥500 mg/kg, the patients were advised to undergo a clinical routine ileocolonoscopy (Fig. 1). At 5-year follow-up, all patients with fecal calprotectin ≥200 mg/kg were asked to send in another stool sample after pausing NSAID medication for 3 weeks. If their fecal calprotectin remained >200 mg/kg when retested, the patients were referred for ileocolonoscopy. Before the ileocolonoscopy, NSAIDs were again paused for 3 weeks. At 5-year follow-up, all ileocolonoscopies were performed by one endoscopist (HS) and followed the same procedure.

### Data analyses

Data from all patients who participated at baseline (*n* = 204) were analyzed with the aim of determining the 5-year incidence of IBD and the predictors thereof. For all other data analyses, only the patients who participated both at baseline and at 5-year follow-up (*n* = 164) were included.

### Statistical analyses

Statistical analyses were performed using IBM SPSS Statistics 23 software (IBM, Armonk, NY, USA). Descriptive statistics are presented as median and IQR. In comparisons between groups, the Mann-Whitney *U* test was used for continuous variables, and the chi-square test or Fisher’s exact test was used for categorical variables. The Wilcoxon signed-rank test was used for repeated measurements. Correlations were calculated using Spearman’s correlation (*r*_s_). Owing to its skewed distribution, fecal calprotectin was log-transformed and used as an outcome in linear regression with a stepwise model. All tests were two-tailed, and *p* ≤ 0.05 was considered statistically significant.

## Results

### Characteristics of the study population

In total, 164 patients, comprising 45% women and 55% men, completed the study and were examined at the two study visits (baseline and 5-year follow-up). The characteristics of the study population are provided in Table 1.

**Table 1:** Characteristics of the 164 patients with ankylosing spondylitis at baseline and 5-year follow-up

	Baseline	5-Year follow-up	*p* Value
Sex, female/male	74 (45)/90 (55)		
Age, years	49.5 (41–62)	54.5 (46–67)	<0.001
Years since onset of AS symptoms	22 (13–34)	27 (18–39)	<0.001
Years since AS diagnosis	12 (5–23)	17 (10–28)	<0.001
HLA-B27-positive	141 (86)		
Fecal calprotectin, mg/kg	86 (45–222)	82 (35–190)	0.660
Fecal calprotectin level			
≤ 50 mg/kg	48 (29.3)	60 (36.6)	0.660
51–199 mg/kg	69 (42.1)	64 (39.0)	
200–499 mg/kg	32 (19.5)	29 (17.7)	
500–999 mg/kg	13 (7.9)	8 (4.9)	
>1000 mg/kg	2 (1.2)	3 (1.8)	
Serum calprotectin, ng/ml	645 (282–705)	No data	N/A
CRP, mg/L	2 (1–6)	3 (1–6)	0.434
ESR, mm/h	11 (7–18)	8 (4–14)	<0.001
ASDAS_CRP_ score	2.2 (1.6–3.0)	2.1 (1.3–2.7)	<0.001
BASDAI score	3.1 (1.6–5.2)	3.2 (1.8–5.2)	0.713
BAS-G score	2.7 (1.2–5.7)	2.8 (1.4–5.9)	0.187
BASFI score	2.3 (1.0–3.8)	2.3 (1.0-4.1)	0.443
BASMI score	2.8 (2.0–4.0)	3.4 (2.4–4.6)	<0.001
Current smoker	15 (9.1)	11(6.7)	0.388
NSAIDs	128 (78)	125 (76)	0.868
Daily use	73 (45)	66 (40)	
As needed	55 (34)	59 (36)	
TNF blockers	33 (20)	39 (24)	0.238
Infliximab	24 (15)	21 (13)	0.664
Adalimumab	4 (2)	8 (5)	0.289
Golimumab	0 (0)	4 (2)	N/A
Etanercept	5 (3)	6 (4)	1.000
DMARDs			
Methotrexate	35 (21)	29 (18)	0.189
Sulfasalazine	16 (10)	8 (5)	0.004
Regular occurrence of gastrointestinal symptoms			
Loose stools	70 (43)	66 (40)	0.401
Mucus in diarrhea	25 (15)	34 (21)	0.405
Blood in stools	22 (13)	21 (13)	1.000
Blood in diarrhea	8 (5)	9 (5)	1.000
Abdominal pain	24 (15)	33 (20)	0.210
Obstipation	54 (33)	41 (25)	0.008
Reflux symptoms	65 (40)	62 (38)	0.391
Epigastric pain	47 (29)	46 (28)	1.000

*Abbreviations: AS* Ankylosing spondylitis, *CRP* C-reactive protein, *DMARD* Disease-modifying antirheumatic drug, *ESR* Erythrocyte sedimentation rate, *ASDAS*_*CRP*_ Ankylosing Spondylitis Disease Activity Score based on C-reactive protein, *BASDAI* Bath Ankylosing Spondylitis Disease Activity Index, *BAS-G* Bath Ankylosing Spondylitis Patient Global Score, *BASFI* Bath Ankylosing Spondylitis Functional Index, *BASMI* Bath Ankylosing Spondylitis Metrology Index, *NSAID* nonsteroidal anti-inflammatory drug, *TNF* tumor necrosis factor

Data are presented as median and [interquartile range] or number (%)

### Intraindividual variation in fecal calprotectin between baseline and 5-year follow-up

Fecal calprotectin was elevated (>50 mg/kg) in 70.7% (116 of 164) of the patients at baseline and in 63.4% (104 of 164) of the patients at 5-year follow-up. The levels of fecal calprotectin did not differ between baseline and follow-up (*p* = 0.660) (Table 1).

**Fig. 2:**
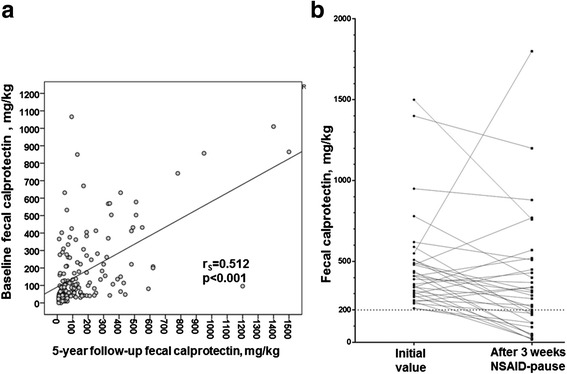
**a** Scatterplot of fecal calprotectin at baseline and 5-year follow-up in 164 patients with ankylosing spondylitis (AS). **b** Fecal calprotectin before and after a 3-week pause of nonsteroidal anti-inflammatory drug (NSAID) use at 5-year follow-up in 33 patients with AS

The change in fecal calprotectin between baseline and the 5-year follow-up was ≤50 mg/ml in 48% of the patients, 51–100 mg/kg in 16%, and 101–200 mg/kg in 16%. The median change between baseline and 5-year follow-up for calprotectin was 1.5 (IQR −52.7 to 52.7) mg/kg. The intraindividual correlation coefficient for fecal calprotectin at baseline and follow-up was *r*_S_ = 0.512 (*p* < 0.001) (Fig. 2a).

### Fecal calprotectin correlates with disease activity and physical function

At both visits, fecal calprotectin was positively and significantly correlated with parameters reflecting higher disease activity and poorer function at the same visit, such as CRP, ESR, ASDAS_CRP_, BASDAI, BASFI, and BASMI. In addition, fecal calprotectin at baseline was positively associated with higher levels of CRP, ESR, ASDAS_CRP_, and BASFI at 5-year follow-up (Table 2).

**Table 2:** Spearman’s correlations between fecal calprotectin and parameters reflecting disease activity and physical function at baseline and 5-year follow-up

	CRP		ESR		ASDAS_CRP_		BASDAI		BASFI		BASMI	
	Baseline	5-year follow-up	Baseline	5-year follow-up	Baseline	5-year follow-up	Baseline	5-year follow-up	Baseline	5-year follow-up	Baseline	5-year follow-up
FC Baseli﻿ne	0.280	0.209	0.222	0.169	0.260	0.240	0.190	N.S.	0.166	0.166	N.S.	N.S.
*p*-value	< 0.001	*p* = 0.007	*p* = 0.004	*p* = 0.031	*p* = 0.001	*p* = 0.002	*p* = 0.016		*p* = 0.034	*p* = 0.034		
FC 5﻿-year	N.S.	0.219	N.S.	0.172	N.S.	0.207	N.S.	N.S.	N.S.	0.154	N.S.	0.207
*p*-value		*p* = 0.005		*p* = 0.027		*p* = 0.008				*p* = 0.048		*p* = 0.008

*Abbreviations: CRP* C-reactive protein, *ESR* Erythrocyte sedimentation rate, *ASDAS*_*CRP*_ Ankylosing Spondylitis Disease Activity Score based on C-reactive protein, *BASDAI* Bath Ankylosing Spondylitis Disease Activity Index, *BASFI* Bath Ankylosing Spondylitis Functional Index, *BASMI* Bath Ankylosing Spondylitis Metrology Index, *FC* fecal calprotectin

The patients who were smoking at baseline (9%) had, in comparison with nonsmokers, significantly higher fecal calprotectin at 5-year follow-up (median [IQR] 107 [43–402] vs. 85 [45–221] mg/kg, *p* = 0.026). Baseline serum calprotectin was also positively correlated with fecal calprotectin, both at baseline (r_S_ = 0.252, *p* = 0.001) and at 5-year follow-up (*r*_S_ = 0.170, *p* = 0.030).

### Fecal calprotectin and medication

**Fig. 3:**
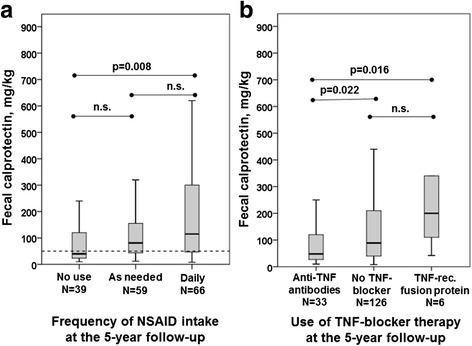
**a** Box plot showing the association between fecal calprotectin and frequency of NSAID intake at 5-year follow-up. **b** Box plot showing the distribution of fecal calprotectin among patients on anti-TNF antibodies (infliximab, adalimumab, golimumab) vs. no TNF blocker therapy vs. TNF receptor fusion proteins (etanercept) at 5-year follow-up. Values are median (*horizontal lines*), IQR (*boxes*), and total range (*whiskers*). *NSAID* Nonsteroidal anti-inflammatory drug, *TNF* Tumor necrosis factor

The medications used at baseline and 5-year follow-up are listed in Table 1. Fecal calprotectin was significantly higher in NSAID users than in nonusers at both study visits (baseline median [IQR] 105 [45–262] vs. 68 [44–93] mg/kg, *p* = 0.032; 5-year follow-up 93 [44–215] vs. 40 [23–120] mg/kg, *p* = 0.011) (Fig. 3a). The users of anti-tumor necrosis factor (anti-TNF) antibodies (infliximab, adalimumab, golimumab) had significantly lower fecal calprotectin than patients without TNF blocker therapy at both visits (baseline 43 [17–84] vs. 99 [54–256] mg/kg, *p* = 0.004; 5-year follow-up 48 [26–135] vs. 89 [40–210] mg/kg, *p* = 0.022), whereas no such difference was found between users of TNF receptor fusion proteins (etanercept) and nonusers of TNF blockers. At 5-year follow-up, the patients treated with TNF receptor fusion proteins had significantly higher fecal calprotectin than the patients treated with anti-TNF antibodies (200 [93–555] vs. 48 [26–135] mg/kg; *p* = 0.016) (Fig. 3b). Comedication with methotrexate and a TNF blocker was common. Methotrexate was mostly taken in low doses (5–15 mg weekly). At 5-year follow-up, the use of methotrexate among patients on anti-TNF antibodies was not significantly different from the use in patients taking etanercept (18 of 33 [54.5%] vs. 2 of 6 [33%]; *p* = 0.407 by Fisher’s exact test).

Although the patients treated with TNF blockers had a significantly lower frequency of NSAID intake than the patients without TNF blockers, the significant differences in fecal calprotectin between users and nonusers of disease-modifying antirheumatic drugs and/or TNF blockers remained after adjustment for NSAID use. Linear regression analysis was performed with log-transformed fecal calprotectin at 5-year follow-up as an outcome measure, and the following baseline values were used as covariates: sex, age, serum calprotectin, fecal calprotectin, CRP, ASDAS_CRP_, BASFI, smoking, and use of NSAIDs and anti-TNF antibodies/TNF receptor fusion proteins. Only fecal calprotectin at baseline (β = 0.001, SE = 0.000, 95% CI 0.0008–0.0015, *p* < 0.001) and smoking at baseline (B = 0.241, SE = 0.121, 95% CI 0.002–0.479, *p* = 0.048) remained independently associated with higher fecal calprotectin at 5-year follow-up (*R* ^2^ = 0.247).

### Gastrointestinal symptoms

A high frequency of gastrointestinal symptoms (diarrhea, presence of blood or mucus in stools, obstipation, abdominal pain, tenesmus, reflux symptoms, and epigastric pain) was reported by the participants both at baseline and at 5-year follow-up (Table 1). There were no significant associations between the levels of fecal calprotectin and gastrointestinal symptoms at any of the time points, however.

### Fecal calprotectin before and after of 3-week NSAID pause

At 5-year follow-up, the patients who had fecal calprotectin ≥200 mg/kg (*n* = 40 [24%]) were asked to send in a new stool sample after pausing NSAID use for 3 weeks, which a total of 33 patients did. After the 3-week NSAID pause, fecal calprotectin dropped by 116 (−20 to 289) mg/kg from a range of 410 (295–510) mg/kg to 290 (120–480) mg/kg (*p* = 0.005) (Fig. 2b).

### Development of gut inflammation during 5 years of follow-up

Ileocolonoscopies were performed in 8 patients at baseline and in 16 patients at 5-year follow-up (Fig. 1). The findings from the ileocolonoscopies are summarized in Table 3. Altogether, 10 (41.7%) of the 24 patients who underwent ileocolonoscopy had inflammatory changes in the gut biopsies. In addition, five patients (20.8%) had only macroscopic changes.

**Table 3:** Development of gut inflammation in 204 patients with ankylosing spondylitis between baseline and 5-year follow-up

Type of inflammation	Baseline	5-Year follow-up	Total
Diagnosis of Crohn’s disease	1	2	3
Diagnosis of ulcerative colitis	0	0	0
Diagnosis of lymphocytic colitis	1	0	1
Subclinical findings in the distal ileum			5
Macroscopic changes: aphthous ulcerations		1	
Microscopic changes: chronic inflammation		1	
Both macroscopic and microscopic changes	2	1	
Subclinical findings in the colon			6
Macroscopic changes: ulcerations	3	1	
Microscopic changes: chronic inflammation		1	
Both macroscopic and microscopic changes		1	
Total			15

At 5-year follow-up, 3 patients (1.5%) were diagnosed with CD (corresponding to an annual incidence of 294 new cases per 100,000 person-years), and 1 patient (0.5%) was diagnosed with lymphocytic colitis. No cases of UC were found. Additionally, five patients (2.5%) demonstrated characteristics of subclinical CD with aphthous ulcerations and/or chronic inflammation in the distal ileum, and another six patients (2.9%) had ulcerations and/or chronic inflammation in the colon. In total, 15 patients (7.4%) demonstrated characteristics of inflammation in the large or small intestine during the 5-year follow-up period.

### Predictors for development of CD

At baseline, the patients who developed CD had, in comparison with the other patients, higher fecal calprotectin (570 [271–570] mg/kg vs. 85 [43–230]; *p* = 0.014) and more often reported having diarrhea containing mucus (3 of 3 vs. 30 of 201; *p* = 0.004). No significant associations were found between the development of CD and the other baseline parameters. A receiver operating characteristic (ROC) curve was created with baseline fecal calprotectin as the test variable and development of CD as the categorical state variable. The area under the curve was 0.913 (95% CI 0.805–1.000; *p* = 0.014). Using a threshold for baseline fecal calprotectin of 266 mg/kg, based on this ROC curve, the sensitivity for development of CD was 100% and the specificity was 78.7%.

## Discussion

In this longitudinal 5-year study of a well-characterized cohort of patients with AS, we followed the intraindividual variation in fecal calprotectin in relation to disease activity and medication, and we determined the incidence and predictors for IBD. Fecal calprotectin was elevated (>50 mg/kg) in approximately two-thirds of the patients at both the baseline and 5-year follow-up visits. The intraindividual variation was moderate; in 80% of the patients, fecal calprotectin changed <200 mg/kg between the two measurement time points. Smoking and elevated fecal calprotectin at baseline were the strongest predictors for high fecal calprotectin at 5-year follow-up. Of the 204 patients included at baseline, 1.5% were diagnosed with CD and 0.5% with lymphocytic colitis at 5-year follow-up. High fecal calprotectin and presence of mucus in diarrhea at baseline were the main predictors of development of CD in this cohort.

Our results indicate that gut inflammation in AS is associated with higher disease activity in rheumatic disease. Increased fecal calprotectin at baseline was associated with higher ASDAS_CRP_, BASDAI, BASFI, CRP, and ESR at 5-year follow-up. Early prospective studies showed a link between active gut inflammation and persistence of joint and spine inflammation as well as more chronic radiographic changes in the sacroiliac joint and spine [18, 27]. Researchers in two cross-sectional studies reported higher fecal calprotectin in AS than in healthy control subjects [28, 29]. One of the studies showed a positive correlation between fecal calprotectin and BASDAI, BASFI, ESR, and CRP [29]. In studies with the Belgian Inflammatory Arthritis and spoNdylitis cohorT (GIANT) of early SpA, the presence of histological gut inflammation was associated with higher BASDAI and higher BASMI and was also linked to a greater degree of bone marrow edema in the sacroiliac joints visualized by magnetic resonance imaging [13, 30]. The longitudinal results of our study support they hypothesis that inflammation in the gut and the musculoskeletal system may be linked. If gut inflammation is the cause or the consequence of locomotor disease is unknown, however, and fecal calprotectin is only a surrogate measure for gut inflammation.

In a recent report based on the GIANT cohort, fecal calprotectin, serum calprotectin, and CRP were also significantly higher in patients with microscopic gut inflammation [31]. We found that serum calprotectin at baseline was positively correlated with fecal calprotectin, both at baseline and at 5-year follow-up, but serum calprotectin was not a significant predictor for the development of CD.

In the present study, the use of anti-TNF antibodies was associated with lower fecal calprotectin, whereas use of TNF receptor fusion proteins was associated with higher levels of fecal calprotectin, at 5-year follow-up. Infliximab and adalimumab, but not etanercept, are efficacious in the treatment of CD and UC [32]. Similarly, in earlier studies, more flares and onset of IBD were observed in patients with AS treated with etanercept than in patients treated with infliximab [33, 34].

The 5-year incidence of CD in the present study was 1.5%, which corresponds to an annual incidence of 294 new cases per 100,000 person-years. This can be compared with the highest reported annual incidence of CD in the general population of 12.7 per 100,000 person years in Europe and 20.2 per 100,000 person-years in North America [35]. Mielants et al. reported an incidence of IBD of 5% in a cohort of 217 patients with SpA or AS during a median follow-up of 5.5 years [19]. Similarly to our study, CD was more common than UC in their study. In contrast to our study, ileocolonoscopy was performed on all patients at inclusion, and 49 patients underwent a second endoscopy. Because patients with fecal calprotectin <200 mg/kg were not examined with endoscopy in our study, cases with IBD despite lower fecal calprotectin may have been missed. Moreover, the earlier studies were performed in the “prebiologic era,” which may have affected the results. Furthermore, a few patients with persistently elevated fecal calprotectin declined ileocolonoscopy.

In our study, elevated fecal calprotectin and presence of mucus in diarrhea at baseline were the only significant predictors of the development of CD. The low number of new cases of CD (*n* = 3) may have affected the robustness of the results and the possibility of finding other significant predictors.

The high use of NSAIDs among patients with AS is a problem when studying gut inflammation. NSAID-induced enteropathy is difficult to distinguish from AS-associated gut inflammation and is associated with increased fecal calprotectin [23]. In the present study, NSAID use was stopped for 3 weeks before retesting fecal calprotectin and before ileocolonoscopies, which resulted in a drop of a median of 116 mg/kg in fecal calprotectin. We found that 3-week cessation of NSAID use before testing fecal calprotectin was a reasonable course of action and acceptable for most patients.

The strengths of the present study are its prospective design with two measurement time points, the relatively large and well-characterized cohort, and cessation of NSAIDs before ileocolonoscopy at follow-up. The study’s weaknesses are that ileocolonoscopy was not performed in all patients, which may have resulted in a lower incidence of IBD. The ileocolonoscopies were also performed at different thresholds of fecal calprotectin at baseline and 5-year follow-up (Fig. 1).

## Conclusions

Fecal calprotectin was elevated in two-thirds of the patients with AS and was positively associated with parameters reflecting higher disease activity and poorer physical function at both study visits. Use of NSAIDs and TNF receptor fusions proteins was associated with higher levels of fecal calprotectin, whereas use of anti-TNF antibodies was associated with lower levels of fecal calprotectin. Further studies are needed to prove whether anti-TNF treatment has an effect on subclinical gut inflammation in AS and whether it can prevent the development of IBD. The 5-year incidence of CD was 1.5%, and elevated fecal calprotectin at baseline was the strongest predictor of the development of IBD. On the basis of these results, we propose that fecal calprotectin may be a potential biomarker to identify patients with AS at risk of developing IBD. Thus, fecal calprotectin quantification should be considered, especially in patients with high disease activity and gastrointestinal symptoms. If possible, NSAIDs should be stopped temporarily before measuring fecal calprotectin to reduce the confounding effect of NSAID enteropathy on the measurement.

## Abbreviations

AS: Ankylosing spondylitis
ASDAS: Ankylosing Spondylitis Disease Activity Score
ASDAS_CRP_: Ankylosing Spondylitis Disease Activity Score based on C-reactive protein
BASDAI: Bath Ankylosing Spondylitis Disease Activity Index
BASFI: Bath Ankylosing Spondylitis Functional Index
BAS-G: Bath Ankylosing Spondylitis Patient Global Score
BASMI: Bath Ankylosing Spondylitis Metrology Index
CD: Crohn’s disease
CRP: C-reactive protein
DMARD: Disease-modifying antirheumatic drug
ELISA: Enzyme-linked immunosorbent assay
ESR: Erythrocyte sedimentation rate
GIANT: Belgian Inflammatory Arthritis and spoNdylitis cohorT
IBD: Inflammatory bowel disease
NSAID: Nonsteroidal anti-inflammatory drug
ROC: Receiver operator characteristic
SpA: Spondylarthritis
TNF: Tumor necrosis factor
UC: Ulcerative colitis
